# Phenotypic Analysis and Molecular Characterization of Enlarged Cell Size Mutant in *Nannochloropsis oceanica*

**DOI:** 10.3390/ijms241713595

**Published:** 2023-09-02

**Authors:** Weinan Xu, Yihua Lin, Yu Wang, Yanyan Li, Hongmei Zhu, Hantao Zhou

**Affiliations:** 1 State Key Laboratory of Marine Environmental Science, Xiamen University, Xiamen 361000, China; xuweinan@stu.xmu.edu.cn (W.X.); linyihua@stu.xmu.edu.cn (Y.L.); wangyuu@stu.xmu.edu.cn (Y.W.); yanyanli2016@stu.xmu.edu.cn (Y.L.); 2 College of Ocean and Earth Sciences, Xiamen University, Xiamen 361000, China; flyzhu324@163.com

**Keywords:** cell cycle, cell division, cell size, bioinformation, *Nannochloropsis*

## Abstract

The cell cycle is the fundamental cellular process of eukaryotes. Although cell-cycle-related genes have been identified in microalgae, their cell cycle progression differs from species to species. Cell enlargement in microalgae is an essential biological trait. At the same time, there are various causes of cell enlargement, such as environmental factors, especially gene mutations. In this study, we first determined the phenotypic and biochemical characteristics of a previously obtained enlarged-cell-size mutant of *Nannochloropsis oceanica*, which was designated *ECS*. Whole-genome sequencing analysis of the insertion sites of *ECS* indicated that the insertion fragment is integrated inside the 5′-UTR of U/P-type cyclin *CYCU;1* and significantly decreases the gene expression of this cyclin. In addition, the transcriptome showed that *CYCU;1* is a highly expressed cyclin. Furthermore, cell cycle analysis and RT-qPCR of cell-cycle-related genes showed that *ECS* maintains a high proportion of 4C cells and a low proportion of 1C cells, and the expression level of *CYCU;1* in wild-type (WT) cells is significantly increased at the end of the light phase and the beginning of the dark phase. This means that *CYCU;1* is involved in cell division in the dark phase. Our results explain the reason for the larger *ECS* size. Mutation of *CYCU;1* leads to the failure of *ECS* to fully complete cell division in the dark phase, resulting in an enlargement of the cell size and a decrease in cell density, which is helpful to understand the function of *CYCU;1* in the *Nannochloropsis* cell cycle.

## 1. Introduction

The cell cycle is a fundamental cellular process in eukaryotes that includes cell growth, DNA replication, and cell division [1]. The cell cycle consists of pre-DNA synthesis (G1), DNA synthesis (S), post-DNA synthesis (G2), and mitosis (M phase). There are two checkpoints (eukaryotes) between the G1/S and G2/M phases: commitment (*Chlamydomonas reinhardtii*; S/M phase) and start (yeast; G1–S phase). These checkpoints are present to ensure cell size and DNA replication readiness [2,3,4]. The cell cycle is vital for microalgae. When microalgae are in a suitable environment, cells accumulate sufficient energy and the population rapidly increases in number after one cell cycle [5].

With the rapid development of omics and molecular biology, we can identify cell-cycle-related genes in different eukaryotes via homology and verify their functions. Specific cyclin and cyclin-dependent kinase (*CDK*) complexes are involved in specific cell cycle phases [4]. Phosphorylation-activated cyclin and *CDK* complexes can phosphorylate retinoblastoma (Rb) protein and release *E2F*/*DP* transcriptional activator to stimulate the expression of downstream S-phase-related genes, which promotes cell entry into the S phase [6]. Although the cell-cycle-related genes in *Chlamydomonas reinhardtii* are conserved [2,7], with more research, researchers have found new regulatory mechanisms. *CDKA1* regulates the cell size commitment and may be required to set a higher-cell-size threshold controlling the transition between nondividing and dividing states. The MAT3/E2F/Dp1 pathway regulates the division number as well as the commitment size, but *CDKA1* and the MAT3-E2F/DP1 pathway influence cell size and commitment in distinct ways [8]. *CDKG1*, a sizer protein that enables mother cells to execute the correct number of mitotic cell divisions according to mother cell size, binds to D-type cyclins and phosphorylates the retinoblastoma (RB) tumor suppressor pathway homolog *MAT3* to regulate mitotic counting [9]. Atkins and Cross proposed a negative feedback loop model based on cyclins A-*CDKA* and B-*CDKB*, and *CDKA* activates cyclin B-*CDKB*; in turn, cyclin B-*CDKB* promotes mitotic entry and inactivates cyclin A-*CDKA* [10]. Similarly, new cell regulatory mechanisms have been found in *Phaeodactylum tricornutum*. *CDKA2* was originally assigned to A-type CDKs, but displays some typical characteristics of plant-specific B-type CDKs and plays an essential role in the G2/M phase [11]. Diatom-specific cyclins respond to nutrient availability [12]. AUREOCHROME1a and the transcription factor *bZIP10* induce *dsCYC2* transcriptional expression under blue light, light-dependent dsCYC2 interacts with *CDKA1* to control the G1/S cell size checkpoint, and *dsCYC2* silencing decreases the rate of cell division in light–dark cycles [13].

The genus *Nannochloropsis* is assigned to the class Eustigmatophyceae, Heterokonta. *Nannochloropsis* species have a high triacylglycerol content, and their polar lipids are rich in eicosapentaenoic acid (EPA), an omega-3 long-chain polyunsaturated fatty acid. With the use of updated genetic engineering toolkits, *Nannochloropsis* has also been considered as a model for the study of microalgal lipid metabolism and as a chassis for synthetic biology [14]. *Nannochloropsis* loses its flagella and mobility because the genome has few flagellum-associated and movement-associated genes. Meanwhile, the absence of meiosis-specific and mating-associated genes leads to the loss of their ability to sexually reproduce [15]. Cell-cycle-related genes of *Nannochloropsis* have been reported [16]; however, the functions of these genes have not been described. The unique evolutionary characteristics of *Nannochloropsis* indicate that the cell cycle may differ from what is known. The phenomenon of cell enlargement is universal in algae research, and dry biomass, lipid, and fatty acid methyl ester (FAME) contents change in terms of cell size [17,18,19]. Meanwhile, the cell size is affected by environmental changes, including phosphorus stress [20,21], nitrogen stress [21], temperature [22], and especially light quality [22,23,24]. Blue light stimulates cell enlargement by delaying DNA replication and mitosis, while red light has the opposite effect [22,25]. In addition, gene mutations lead to cell enlargement, including UV mutagenesis [17], laboratory adaptation evolution [18], natural modification under laboratory conditions [19], and artificial selection [26]. Such phenotypic traits are caused by multigene mutations according to genome sequencing analysis [19]. Thus, the cell size regulators for *Nannochloropsis* are still unclear, and whether having a large cell size affects the photosynthetic characteristics, nutrient storage, and potential molecular mechanism of the large-cell-size phenotype has yet to be explored.

In this study, we used previously obtained *ECS*, which is a randomly inserted mutant whose genome contains the exogenous resistance gene (*Ble*). *ECS* was selected according to the FSC value (indicated cell size) via flow cytometry, and it has a large-cell-size phenotype. Thus, we first studied the phenotypic and biochemical characteristics of *ECS*. Furthermore, to analyze the phenomenon of cell enlargement, we integrated whole-genome sequencing analysis of insertion sites, an analysis of gene families and publicly available transcriptional data, flow cytometry analysis of the cell cycle, and real-time reverse transcription quantitative (RT-qPCR) analysis of cell-cycle-related genes. It was found that mutation of *CYCU;1* caused *ECS’s* failure to fully complete cell division in the dark phase, resulting in a larger size and altering the expression level of cell-cycle-related genes. Therefore, our results explain the reason for *ECS* mutant cell enlargement and reveal that *CYCU;1* regulates cell division, and thus controls cell size and cell density, which provides a target gene for genetic engineering to regulate microalgae cell size and density.

## 2. Results

### 2.1. Cell Density and Cell Size in ECS

**Figure 1:**
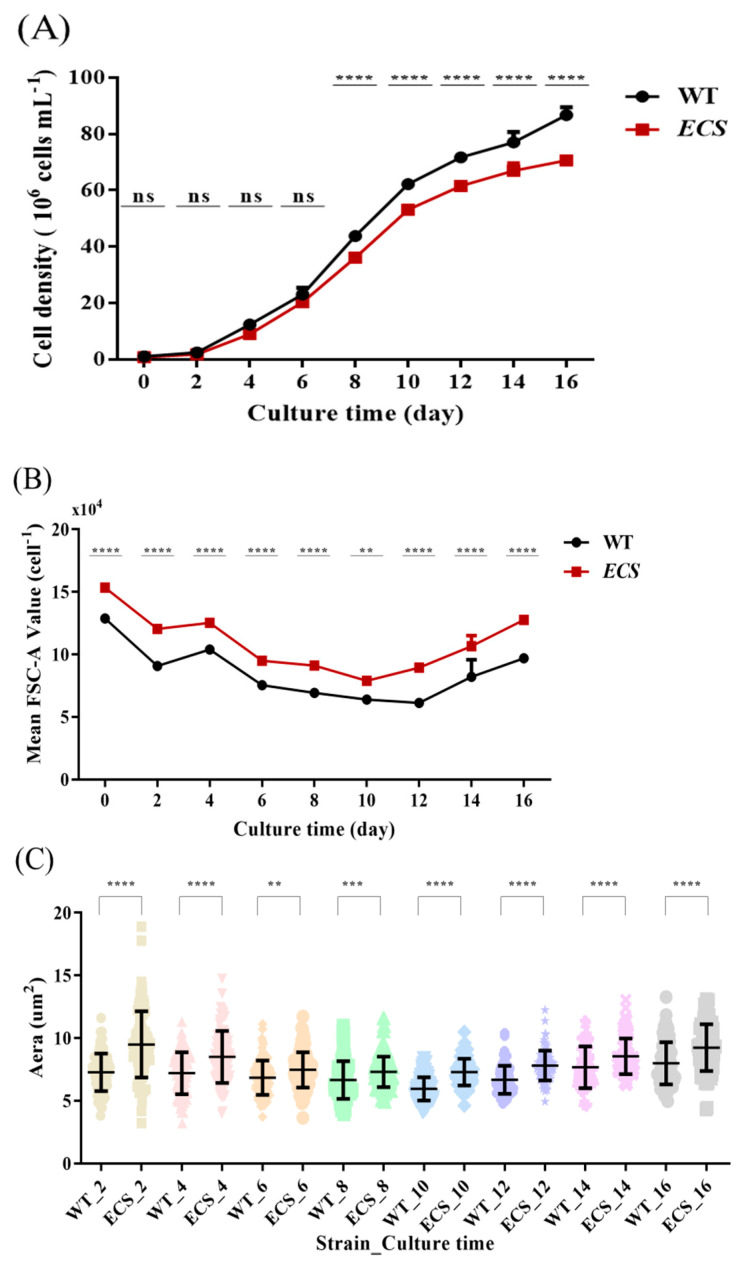
Phenotypes of WT and *ECS*. (**A**) Growth curve of WT and *ECS* after inoculation. (**B**) Flow cytometry signal of mean forward scatter per cell. (**C**) Statistical analysis of cell area through fluorescence microscopy. ns indicates *p* > 0.05, ** *p* < 0.01, *** *p* < 0.001, **** *p* < 0.0001. Error bars indicate standard deviations. Cell density and mean FSC-A values were analyzed using two-way ANOVA (GraphPad Prism), while area was analyzed through *t*-test (GraphPad Prism).

Based on the growth curve, we observed that the growth of *ECS* was much slower than that of its wild type after culture to the middle logarithmic phase (Figure 1A). On day 8, the WT cell density was 4.3 × 10^7^ cells mL^−1^, while that of *ECS* was 3.6 × 10^7^ cells mL^−1^, and the latter was significantly lower than the former after day 8, decreasing by 17.4%, 14.6%, 14.2%, 13.1%, and 18.6%. In addition, we also noted changes in cell size, with *ECS* being significantly larger than WT (Figure 1B,C). *ECS* had a significantly increased cell size compared to WT at different time points based on an analysis of the forward scatter area (FSC-A) signal via flow cytometry, with an increased range of 19.3% to 45.9% (*p* < 0.01) and an average increase of approximately 29%. It was also found that *ECS* was larger than WT at each time point through fluorescence microscopy. The cell area of *ECS* increased significantly compared to WT, increasing from 5.9% to 30.6% (*p* < 0.01; Supplementary Table S2 and Figure S1), and the average increase was approximately 16.1%.

### 2.2. Chlorophyll Fluorescence and Chlorophyll Content in ECS

**Figure 2:**
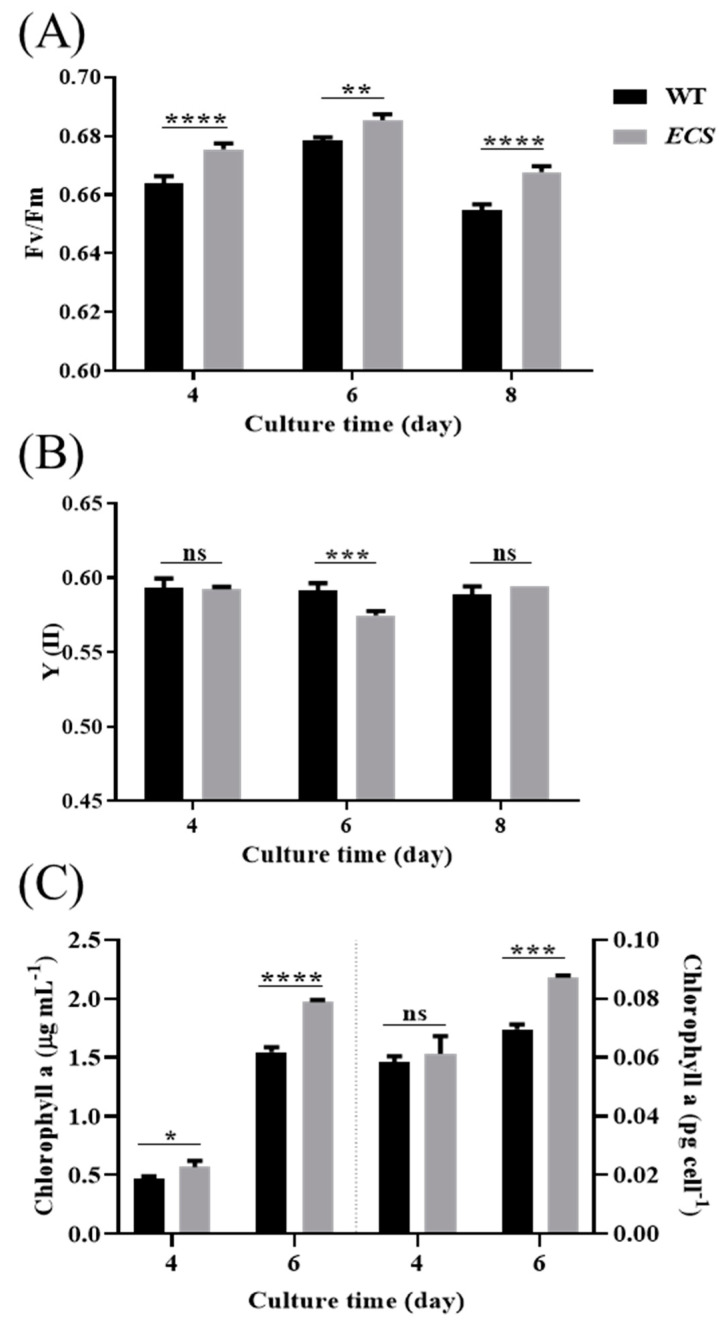
Chlorophyll fluorescence and chlorophyll content of WT and *ECS*. (**A**) Maximum PSII quantum yield (Fv/Fm). (**B**) Effective quantum yield of PSII (YII). (**C**) Chlorophyll a content per unit volume and per cell. ns indicates *p* > 0.05, * *p* < 0.05, ** *p* < 0.01, *** *p* < 0.001, **** *p* < 0.0001. Error bars represent standard deviations calculated from three independent biological replicates. All data were analyzed through two-way ANOVA (GraphPad Prism).

We found that the Fv/Fm was significantly higher for *ECS* than WT (Figure 2A), but Y (II) was significantly lower on day 6 (Figure 2B). The chlorophyll a content per milliliter was significantly higher for *ECS* than WT, and the cell level was significantly higher on day 6 (Figure 2C). 

### 2.3. Dry Biomass, Protein, Total Carbohydrate, and Lipid Contents in ECS

**Figure 3:**
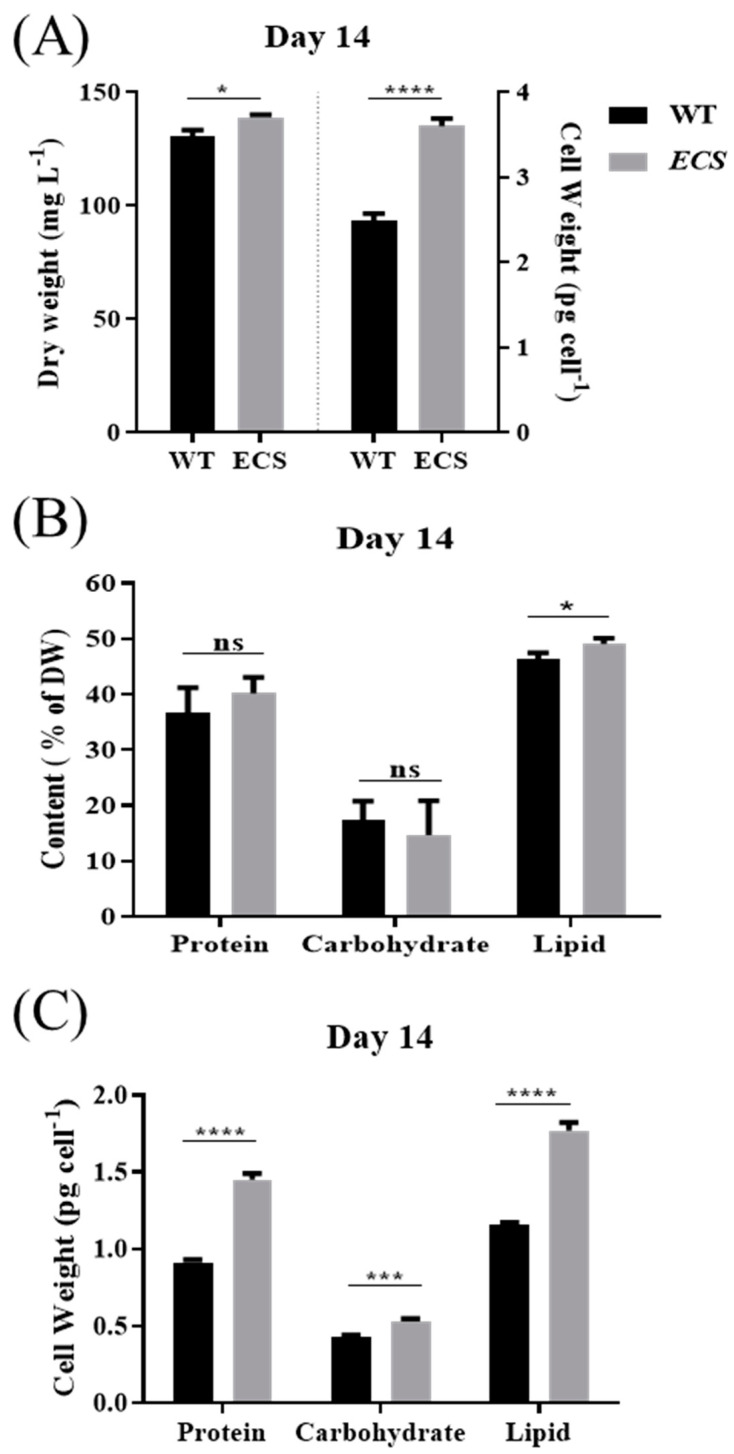
Dry biomass, protein, carbohydrate, and lipid contents of WT and *ECS*. (**A**) Dry weight and dry weight per cell on day 14. (**B**) Protein, carbohydrate, and lipid contents on day 14. (**C**) Protein, carbohydrate, and lipid contents per cell on day 14. ns indicates *p* > 0.05, * *p* < 0.05, *** *p* < 0.001, **** *p* < 0.0001. Error bars represent standard deviations calculated from three independent biological replicates. All data were analyzed through *t*-test (GraphPad Prism).

In addition, we analyzed the biomass accumulation of *ECS* and WT and found that *ECS* increased by approximately 6.5% per unit volume compared with WT and the cell level increased by approximately 45% (*p* < 0.0001) (Figure 3A), and the protein and lipid contents were also higher, increasing by approximately 9.5% (*p* > 0.05) and 5.8% (*p* < 0.05), respectively (Figure 3B). It is worth noting that the protein, carbohydrate, and lipid contents per cell were significantly higher in *ECS* than in WT, especially the protein and lipid contents, which increased by 58.8% (*p* < 0.0001) and 53.5% (*p* < 0.0001), respectively (Figure 3C).

### 2.4. Insertion Is Identified to Be in Locus NO14G02130, Encoding a Cyclin Protein

**Figure 4:**
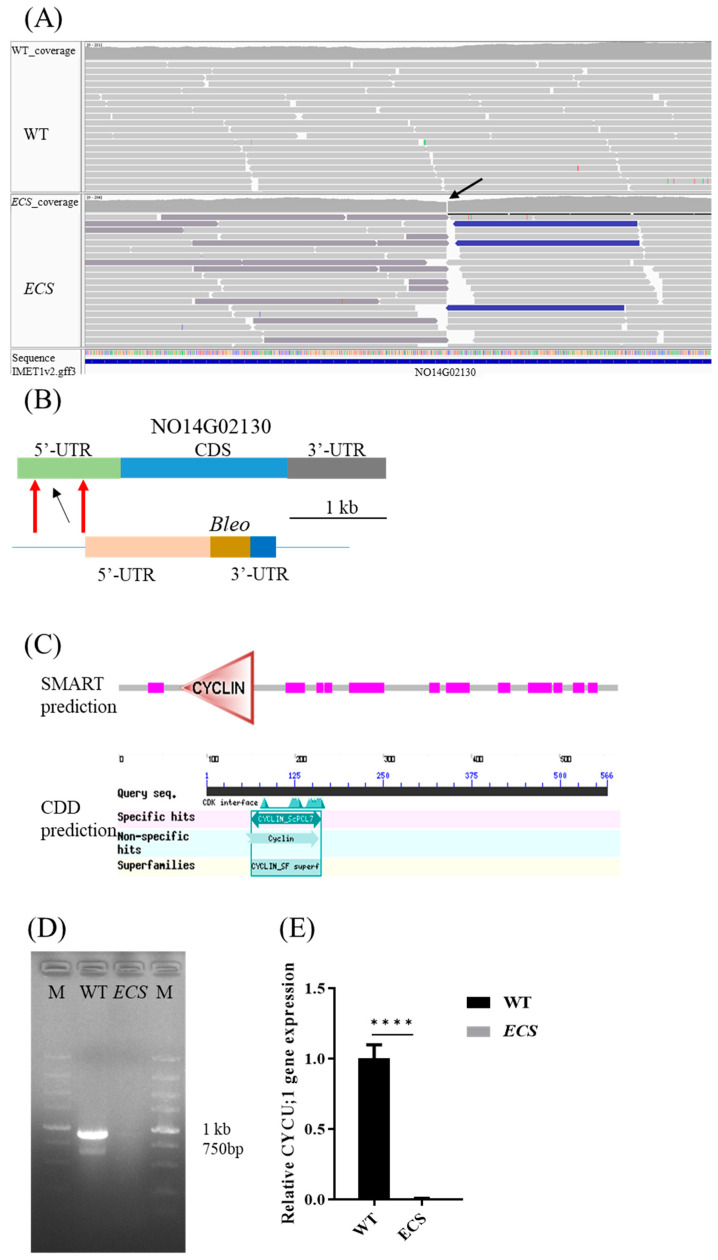
Identification and confirmation of insertion sites of WT and *ECS*. (**A**) Visualization of whole-genome sequencing analysis of insertion site by IGV. (**B**) Schematic diagram of insertion site. (**C**) Gene annotation of NO14G02130 using SMART and CDD. (**D**) Identification of inserted fragment through PCR amplification. (**E**) Gene expression levels of *CYCU;1* between WT and *ECS* determined through RT-qPCR. Arrows indicate insertion sites. M indicates 5000 marker, **** *p* < 0.0001. *UBCE* gene as internal control and RT-qPCR were analyzed through *t*-test (GraphPad Prism).

Analysis of whole-genome sequencing of *ECS* showed that the insertion fragment was integrated inside the 5′-UTR of NO14G02130 at 643,397 of chromosome 14, which is −871 bp from the ATG start codon (Figure 4A,B). According to conserved domain prediction analysis using SMART and CDD prediction, NO14G02130 encodes a cyclin protein (Figure 4C) and has high amino acid sequence similarity with the *Nannochloropsis gaditana* cyclin-dependent protein EWM24594.1 (E-value = 3 × 10^116^, 52.58% identity on 74% sequence coverage using BLASTP). The fragment was successfully integrated into the genome (Figure 4D), and *CYCU;1* was significantly downregulated (*p* < 0.0001, Figure 4E).

### 2.5. Identification of Cyclin Gene Family in Nannochloropsis oceanica

To identify the cyclin genes in *N. oceanica* IMET1 proteins, the *N. oceanica* IMET1 protein sequences were subjected to hmmsearch and BLASTP analysis using the Pfam database and the five species cyclin genes. A total of 19 cyclin genes were identified in *N. oceanica* IMET1 (Table 1), all including at least one cyclin domain (PF00134, PF02984, or PF08613). There are 2, 3, 2, 1, 4, 1, 4, and 2 members in the A-, B-, D-, H-, L-, T-, L-, P-, and U-type cyclins, respectively. Furthermore, we found that 6 genes contained both N- and C-domains, 10 genes contained N-domains, and 3 genes contained cyclin domains. The cyclin genes were located on 15 of 30 chromosomes; in particular, chromosomes 2, 3, and 12 had more than 2 cyclin genes, while the others had only 1 cyclin gene. The number of introns in cyclin genes ranged from 0 to 10. The length of cyclin proteins ranged from 310 to 843 amino acids (aa), and proteins ranged in size from 34 to 89 kDa. All analysis results of cyclin genes are presented in Table 1.

### 2.6. Motif Analysis and Light/Dark Transcriptomic Analysis of Cyclin Genes

**Figure 5:**
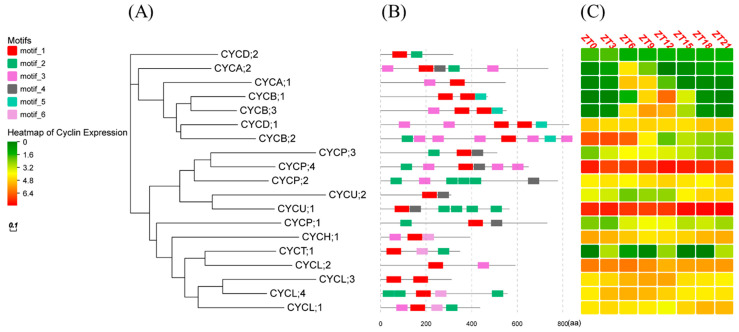
Cyclin gene families of *N. oceanica* IMET1. (**A**) Phylogenetic tree was constructed based on full-length sequences of cyclin proteins. (**B**) Distribution of conserved protein motifs. (**C**) Heatmap of cyclin expression under light/dark cycles based on FPKM value. ZT0, ZT15, ZT18, and ZT21 correspond to dark phase; ZT3, ZT6, ZT9, and ZT12 correspond to light phase.

The *N. oceanica* IMET1 phylogenetic tree was constructed using the maximum-likelihood approach (Figure 5A). The 19 cyclin genes were divided into three clusters: cluster 1 contained A-, B-, and D-type cyclins; cluster 2 contained P- and U-type cyclins; and cluster 3 mainly contained H-, L-, and T-type cyclins. Based on the results of the MEME algorithm, all cyclin genes contained at least one conserved cyclin motif (Figure 5B, Supplementary Table S3). The *CYCU;1* gene had 1 motif 1, 4 motif 2, and 1 motif 4. To understand the expression of cyclins under light/dark conditions, we downloaded the light/dark transcriptome from NCBI. According to our analysis results, there was an oscillatory expression of cyclins; some cyclins were highly expressed in the light phase, such as *CYCA;1* (ZT6), *CYCB;1* (ZT12), and *CYCB;3* (ZT9); and some were highly expressed in the dark phase, such as *CYCL;1* (ZT18), and *CYCU;2* (ZT21); however, *CYCU;1* and *CYCP;4* were highly expressed at different times of the day (Figure 5C, Supplementary Table S4), which suggests that these two cyclins may play significant roles in the cell cycle.

**Figure 6:**
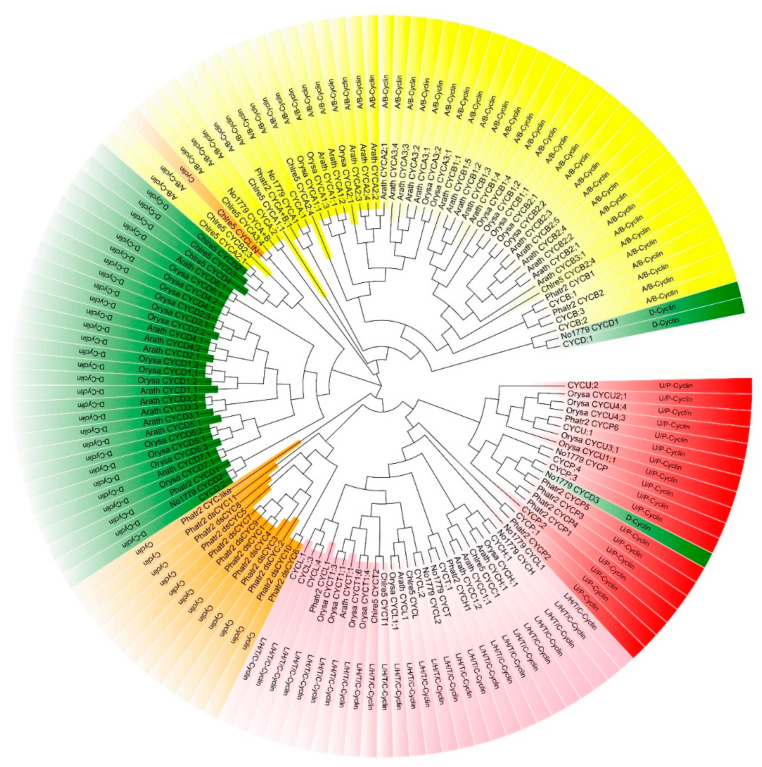
Phylogenetic tree analysis of cyclins in *A. thaliana*, *O. sativa*, *N. oceanica* IMET1, *N. oceanica* CCMP1779, *C. reinhardtii*, and *P. tricornutum*. Tree was divided into five clusters indicated by different colors.

A phylogenetic study was performed using the maximum-likelihood tree approach to understand the evolutionary relationship of *N. oceanica* IMET1 cyclins with several *Nannochloropsis* species: *N. oceanica* CCMP1779 (No1779), Ochrophyta *P. tricornutum* (Phatr2), Chlorophyta *C. reinhardtii* (Chlre5), monocot *O. sativa* (Orysa), and eudicot *A. thaliana* (Arath). The tree was built using 19, 10, 24, 14, 34, and 36 cyclin genes from *N. oceanica* IMET1, *N. oceanica* CCMP1779, *P. tricornutum*, *C. reinhardtii*, *O. sativa*, and *A. thaliana*, respectively. The tree was divided into five clusters (Figure 6), including A/B-type, D-type, L/H/T/C-type, and U/P-type cyclins and cyclins. The U/P-type cyclin cluster contained *O. sativa*, *P. tricornutum*, and *N. oceanica*. On the one hand, *CYCP5* and *CYCP6* of *P. tricornutum* were considered to be markers of the G1 phase [27]. On the other hand, six P-type cyclins of *P. tricornutum* are thought to play a role in phosphate signaling because they are clustered with the PHO80-like proteins [12]; in addition, *OsCYCP1;1*, *OsCYCP4;1*, *OsCYCP4;2*, and *OsCYCP4;3* of *O. sativa* respond to Pi starvation stress, and it was found that overexpression of Pi-starvation-induced *OsCYCP4* interferes with the interaction of OsCYC and OsCDK [28,29], indicating that U/P-type cyclins of *N. oceanica* may be involved in phosphate signaling.

### 2.7. Failure of ECS to Fully Complete Cell Division in the Dark Phase

**Figure 7:**
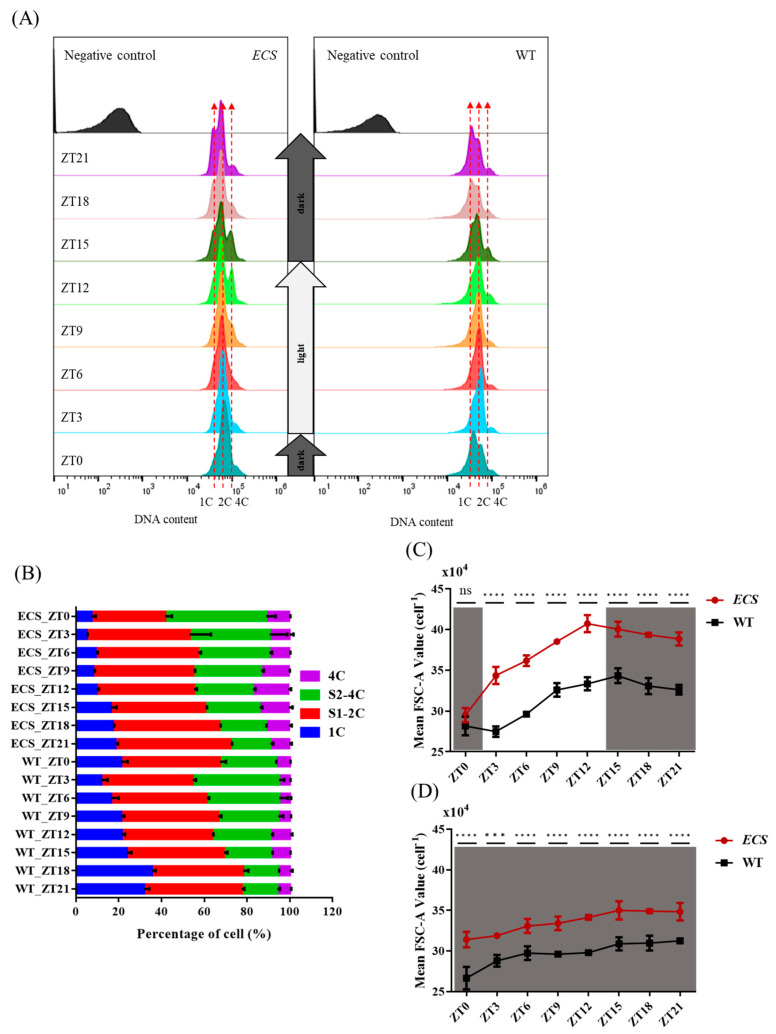
Flow cytometry analysis of cell cycle. (**A**) DNA content per cell measured as DAPI fluorescence using flow cytometry at different times of day (ZT, hours after lights on). (**B**) Estimated percentage of cells in 1C (unreplicated haploid genome), S1 (first round of DNA synthesis), 2C (replicated haploid genome), S2 (second round of DNA synthesis), and 4C (cells with four haploid genomes). (**C**) Flow cytometry signal of mean forward scatter per cell at different times of day. (**D**) Flow cytometry signal of mean forward scatter per cell with an additional 24 h of culturing in the dark after sampling at the same time point. ns indicates *p* > 0.05, *** *p* < 0.001, **** *p* < 0.0001. All data were analyzed using two-way ANOVA (GraphPad Prism); error bars indicate standard deviations calculated from three independent biological replicates.

Flow cytometry was used to observe the signal intensity of DAPI and FSC-A; WT and *ECS* cell growth and DNA replication were observed in the light phase, and cell division was observed in the dark phase (Figure 7). At ZT0, the main peak of WT was located between the 1C and 2C peaks, and the main peak of *ECS* was located at the 2C peak. Partial cells of *ECS* completed the second round of DNA synthesis, and the 4C peak was obvious at ZT12, while the 4C peak of WT had not yet formed, and it was lower in height than that of *ECS*. *ECS* and WT still retained the 2C and 4C peaks when entering the dark phase, and with time, the 4C peaks of both *ECS* and WT gradually decreased. It is noteworthy that when entering the dark phase, the 2C peak of WT shifted to the 1C peak; however, *ECS* remained at the 2C peak and was higher than that of WT (Figure 7A). These results indicate that *ECS* may have failed to fully complete cell division in the dark phase, causing the main peak of *ECS* to be close to 2C at ZT0; thus, *ECS* could complete the second round of DNA synthesis to form the 4C peak earlier.

The cells were divided into different states based on DNA content. We observed that the proportion of 4C cells in *ECS* and WT gradually increased in the light phase, reaching the highest at ZT12, at 16.57% and 9.07%, respectively, and the proportion of 4C cells gradually decreased in the dark phase, and were 8.81% and 5.91%, respectively, at ZT21. The proportion of 1C cells in *ECS* and WT showed a gradually increasing trend, at 18.43% and 31.83% at ZT21, respectively. The proportion of S2-4C cells gradually decreased, which may have converted into 4C cells or undergone direct cell division. The proportion of S1-2C cells was relatively stable in WT, varying from 42.17% to 46.13%. S1-2C cells in *ECS* varied between 44.07% and 49.7%, except at ZT0 and ZT21, when they were 34.43% and 59.93%, respectively (Figure 7B). These results show that *ECS* may be unable to fully complete cell division in the dark phase (Figure S2), and as a result, it always maintains a high proportion of 4C cells and a low proportion of 1C cells.

The cell size changes of *ECS* and WT during light/dark cycles were analyzed using the mean FSC-A signal value. We found that the cell size of *ECS* and WT gradually increased in the light phase, reached a maximum at ZT12, and gradually decreased in the dark phase. The mean FSC-A of *ECS* at ZT15 was 40.05 × 10^4^, while the mean FSC-A of WT was 34.34 × 10^4^, and *ECS* increased by 16.62% compared to WT (Figure 7C). With an additional 24 h of culturing in the dark after sampling at the same time point, the mean FSC-A value of *ECS* was 35 × 10^4^ at ZT15, while that of WT was 30.89 × 10^4^ (Figure 7D).

### 2.8. RT-qPCR Analysis of Cell-Cycle-Related Genes

**Figure 8:**
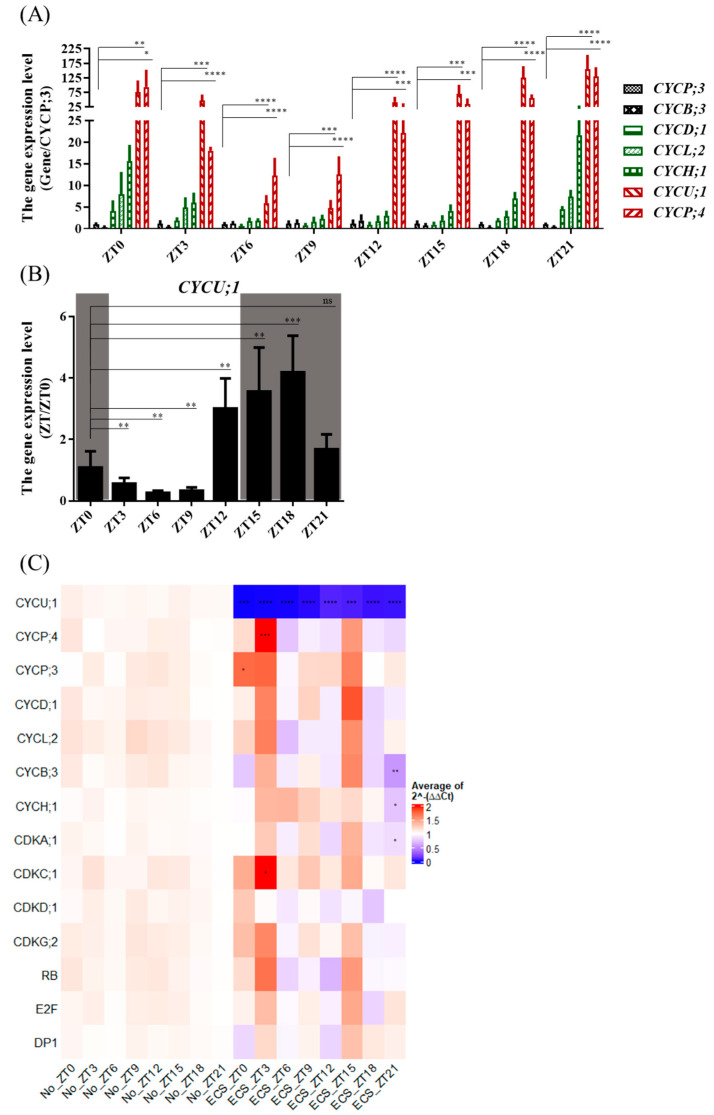
Expression levels of cell-cycle-related genes between WT and *ECS*. (**A**) Expression levels of cyclin genes of WT at different times of day compared with *CYCP;3*. (**B**) Gene expression levels of *CYCU;1* of WT at different times of day compared with ZT0. (**C**) Heatmap of expression of cell-cycle-related genes at different times of day based on average using 2^−ΔΔCT^ method. * *p* < 0.05, ** *p* < 0.01, *** *p* < 0.001, **** *p* < 0.0001. RT-qPCR data are from three independent biological replicates, and error bars indicate standard deviations. *UBCE* gene as an internal control and RT-qPCR were analyzed through *t*-test (GraphPad Prism).

According to the light/dark cycle transcriptomic data analysis, we selected the low expression levels of *CYCP;3* and *CYCB;3*; the medium expression levels of *CYCD;1*, *CYCL;2*, *CYCH;1*, and *CYCP;2*; and the high expression levels of *CYCP;4*, *CYCU;1*, and *CYCP;3* as controls. We observed higher expression levels of *CYCP;4* and *CYCU;1* than the other cyclins at each time point (Figure 8A), consistent with the results of transcriptomic data analysis (Figure 5C). The expression levels of *CYCU;1* and *CYCP;4* were 4.2- to 153.7-fold and 11.1- to 128.2-fold higher, respectively, than that of *CYCP;3*.

Furthermore, we found that, compared to ZT0, the expression levels at the last time point of the light phase (ZT12) and the first two time points of the dark phase (ZT15 and ZT18) were significantly increased by 177%, 228%, and 285%, respectively. The expression levels at the first three time points of the light phase (ZT3, ZT6, and ZT9) were significantly decreased by 47.6%, 75.3%, and 69.1%, respectively, compared with ZT0 (Figure 8B).

To analyze the expression of cell-cycle-related genes of *ECS*, we drew a heatmap of the gene expression levels based on the average 2^−ΔΔCT^ method. *ECS* shows an increased expression of cycle-related genes at time points ZT3 and ZT15 and a decreased expression at time points ZT18 and ZT21 compared with WT (Figure 8C). *CYCP;3* at ZT0 and *CYCP;4* and *CDKC;1* at ZT3 were significantly increased, and *CYCB;3*, *CYCH;1*, and *CDKA;1* at ZT21 were significantly decreased.

These results indicate that *CYCU;1* and *CYCP;4* were the most highly expressed cyclins at different times of the day. The expression levels of *CYCU;1* at ZT12 in the light phase and ZT15 and ZT18 in the dark phase were significantly increased, indicating that *CYCU;1* is involved in cell division in the dark cycle. Mutation of *CYCU;1* induced *CYCP;3* and *CYCP;4* to significantly upregulate their expression to compensate for its missing function, and the expression level of *CDKA;1* was significantly decreased, which affected cell cycle progression.

## 3. Discussion

### 3.1. Cell Size Affects Photosynthetic Characteristics and Nutrient Storage

Internal and external factors, such as genome, nutrients, and growth factors, determine cell size, which affects cellular signaling pathways and metabolic activity [30]. In our study, *ECS*, which was enlarged as a result of the mutation of *CYCU;1*, had increased chlorophyll a and Fv/Fm contents and decreased Y (II) after cell enlargement, which was probably caused by the package effect [31]. Thus, photosynthetic characteristics were correlated with cell size. Malerba et al. used *Dunaliellla teriolecta* as experimental material and obtained algal cells with different sizes through an artificial selection approach. They found that larger sizes led to reduced oxygen production per chlorophyll molecule; increased oxygen production, Fv/Fm, and photosynthetic pigment; and smaller light-harvesting antennae [26]. They also found that larger sizes led to an upregulation of CO_2_-concentrating mechanisms (CCMs), which improved the DIC uptake and led to faster growth and higher maximum biovolume density [32]. In experiments with different light qualities, it was also found that photosynthetic characteristics changed after cell enlargement, including an increase in chlorophyll content, especially the expression of photosynthesis-related genes [24]. In addition, larger cells achieved a balance between growth and photoprotection by sacrificing the growth rate when exposed to strong light [33]. Stephen et al. proposed that the downregulation of cell size maximizes light absorption under limited N to enable the large-scale production of algal oil in continuous output [34]. This means that cell size impacts photosynthetic characteristics. 

Nutrient storage was correlated with cell size. In our study, the total biomass of *ECS* was slightly increased; however, the cell dry weight, protein content, and lipid content per cell were significantly increased (Figure 3C). Similarly, enlarged cell size in *Parachlorella* sp. through adaptive laboratory evolution under salt stress increased fatty acid (FA) and FAME contents and FA productivity and decreased biomass productivity [18]. The combined conditions of blue light and a temperature of 24 or 28 °C induced *C. reinhardtii* to have a large cell size, resulting in the highest protein content; on the contrary, the combined conditions of red-orange light and a temperature of 24 °C promoted carbohydrate content [22]. However, the two smaller-size mutants of *Chlorella vulgaris* were isolated through UV-C irradiation. They showed a significant decrease in biomass and a significant increase in cell concentration and lipid and triacylglycerol (TAG) contents [17]. This means that changes in cell size lead to changes in nutrient storage. Whether cell size can be regulated to obtain specific bioproducts is worth further research. 

### 3.2. Cyclins Respond to Environmental Signals

Cyclins not only activate their CDK partners but also respond to environmental signals: Cyclins respond to light. *dsCYC2*, *CYCP5*, and *CYCP6* respond to the dark–light transition in *P. tricornutum* [12,35] and the PHO80-like cyclin PC3933011 of *Porphyridium cruentum* [36]. Cyclins respond to silica availability, and *dsCYC9* transcript levels were higher in cultures grown in the presence of silica than in those grown without silica [37]. Cyclins respond to phosphates. In our experiments, P/U-type cyclins of *Nannochloropsis* were divided into the same cluster as *O. sativa* and *P. tricornutum* based on the phylogenetic tree (Figure 6). Meanwhile, P/U-type cyclins are homologous with yeast *PHO80* [38]; thus, these cyclins are thought to be involved in phosphate signaling [12,28,29]. *OsCYCP1;1* could partially restore the phosphate signaling pathway in the yeast *pho80* mutant [29], and *SiPHO80* of *Serendipita indica* was upregulated in high-phosphate conditions and also restored Pi homeostasis of the yeast *pho80* mutant [39]. We supposed that *CYCU;1* was responsive to phosphate; hence, we also analyzed the mRNA levels of cyclins based on the published phosphate deprivation transcriptome and found that *CYCU;1* and *CYCP;4* still had the highest cyclin expression under phosphate stress, while the expression of *CYCU;1* was significantly lower in PD48h than in PD0h (log2FC = −0.49, *p* < 0.01). Moreover, it was also reported that the expression level of U/P-type cyclins was stable mainly due to the rich inorganic phosphorus in f/2 medium [40]. Interestingly, by analyzing the mRNA levels of *CYCU;1* under different experimental conditions, we found that *CYCU;1* exhibited high cyclin expression (Supplementary Table S4), indicating that it is an important cyclin for *Nannochloropsis*. Whether *CYCU;1* is involved in the phosphate signaling pathway and its interacting proteins remains to be validated.

### 3.3. Cyclins Play a Role in Cell Division

The cell cycle is a regulatory network composed of a series of cell-cycle-related regulatory genes, in which cyclins are involved in the whole process, especially cell division. In this study, although all cyclins were found to have at least one cyclin box, only *CYCU;1* had the CD20558 structural domain through CDD prediction (Table 1), and it contained Y(L/A)(E/A)RI(F/A)(R/K)(Y/F) and (N/S)VHRLL(V/I)T motifs [29,41]. In *Arabidopsis*, CYCPs may be involved in cell division, cell differentiation, and the nutritional status of the cell by interacting with *CDKA1* [42]. *CYCP2;1* is transcriptionally activated by carbohydrate signals, and it interacts with three of the five mitotic CDKs to promote the G2-to-M transition of cell cycle progression [43]. In rice, overexpressed *OsCYCP4* induced by phosphate induction could compete with the other cyclins for binding with CDKs, and suppress growth by reducing cell proliferation [28]. *OsCYCP3;1* is specifically expressed in the root meristem epidermis and lateral root cap; it regulates meristem cell division by associating with and activating *CKDB2;1* [41]. In addition, different types of cyclins have been reported to be involved in cell division, including A-type, B-type, and D-type cyclins.

In microalgae, *Chlamydomonas CYCA* regulates the timing of cell division [10], *CYCB1* is required for spindle formation, and *CYCB1* is synthesized before each division in the multiple fission cycle and then is rapidly degraded before division occurs. *CYCB1*/*CDKB1* and *APC* modulate microtubule function and assembly while regulating mitotic progression [44]. *CDKG1* binds to D-type cyclins and phosphorylates *MAT3* to regulate mitotic counting [9]. *CYCB1*, *CYCB2*, *dsCYC3*, and *dsCYC4* of *P. tricornutum* are expressed at the G2/M phase, and *CYCB1* is a mitotic biomarker cyclin [12,27]. In plants, D-type cyclins are crucial for growth and development and regulate the cell division process through *CDK*-*CYCD*. Overexpression of the *Populus* D-type cyclin *PsnCYCD1;1* gene in *Arabidopsis* can promote cell division and lead to small cell generation [45], and *PsnCYCD1;1* overexpression in plants could accelerate cell division, causing the generation of small cells and severe morphological changes in the vascular bundles [46]. Overexpression of *PtoCYCD3;3* increases the thickness of secondary xylem and phloem by increasing cambium cell activity, and it may interact with 12 PtoCDK proteins to regulate cell cycle programming [47]. Anantha et al. used overexpressing lines of *PpCYCD1*, *PpCDKA2*, *PpCYCD2*, and *PpCDKA1*, and the phenotypic data confirmed their controlled G1 to S and G2 to M transitions; it was also found that overexpression of *PpCYCD1* or *PpCDKA2* led to larger gametophytes [48]. Meanwhile, the Arabidopsis *CYCD4;2* gene has a promotive function in cell division by binding and activating *CDKA;1* even if it lacks the Rb-binding motif and the PEST sequence, and overexpressing plants showed faster callus formation in a medium containing lower concentrations of auxin [49].

In this study, we observed that the main peak of ECS was maintained at 2C throughout an additional 24 h of dark culture (Figure S2), which suggests that *ECS* cell division was affected. Furthermore, RT-qPCR demonstrated that the expression level of *CYCU;1* significantly increased at the end of the light phase and the beginning of the dark phase (Figure 8B), indicating that the mutation of *CYCU;1* caused incomplete cell division of *ECS* in the dark phase, resulting in lower cell density and larger cells. In addition, in our study, *ECS* downregulated the expression level of *CDKA;1*. Whether or not *CDKA;1* partners with *CYCU;1* to regulate cell division is worth further research.

Cell size plays an essential role in cell division, especially in microalgae with multiple divisions, and the threshold cell size determines the number of successive cell divisions [50,51]. The current understanding is that the controlling cell division model includes timers, sizers, and adders [52]. Our study shows that *ECS* is larger during cell division in the dark phase (Figure 7C,D), but whether this indicates a high threshold for cell division requires further verification. In addition, *Nannochloropsis* is a chassis organism of synthetic biology, and how to increase cell growth is an important research topic. *CYCU;1* provides a target gene for genetic engineering to regulate microalgae cell size and cell density.

## 4. Materials and Methods

### 4.1. Microalgal Strain and Culture Conditions

The microalgal strain used in this study, *N. oceanica* IMET1, was a kind gift from Danxiang Han (Institute of Hydrobiology, Chinese Academy of Sciences). *ECS,* a randomly inserted mutant whose genome contains the exogenous resistance gene (*Ble*), was obtained through electroporation, and was selected according to the FSC value (indicating cell size) via flow cytometry. This means *ECS* possesses a large-cell-size phenotype. In addition, *ECS* is resistant to Zeocin, and the screening concentration was 1 μg mL^−1^.

Microalgae were inoculated into f/2 medium of artificial seawater (S9983, Sigma-Aldrich, St. Louis, MO, USA) supplemented with nutrient enrichment (G0154, Sigma-Aldrich, USA). The cells were cultivated in an artificial climate incubator provided with LED light of 50 μmol photons m^−2^ s^−1^ under a 12/12 h light/dark photoperiod at 25 °C.

### 4.2. Measurement of Growth and Cell Size

Measurement of cell density and cell size: The growth curve of microalgae was determined using a CytoFLEX S flow cytometer (Beckman, Brea, CA, USA) with an initial concentration of 10^6^, and cells were collected at 2-day intervals. The cells were collected and analyzed through flow cytometry to measure cell size (channel FSC-A, forward scatter area). Cell density and mean FSC-A data were analyzed through two-way ANOVA (GraphPad Prism). The cells were measured using fluorescence microscopy (DM6 B, Leica, Wetzlar, Germany); 100 cell areas were calculated using Python scripts, and cell area data were analyzed using *t*-test (GraphPad Prism).

### 4.3. Determination of Chlorophyll Content and Chlorophyll Fluorescence

Determination of chlorophyll content: Chlorophyll a in fresh cells (3 mL culture) was extracted overnight with 3 mL of pure methanol at 4 °C. Before measurement, samples were centrifuged at 4000 rpm for 10 min, and the absorbance of the supernatant was measured using a scanning spectrophotometer (TU-1810, PERSEE, Beijing, China). The Chl a concentration was calculated using the following equation: Chl a (μg mL^−1^) = 16.29 × (A665 − A750) − 8.54 × (A652 − A750) [53].

Chlorophyll fluorescence measurement: The maximum quantum yield of PSII (Fv/Fm) and the effective quantum yield of PSII (Y(II)) were assessed using a pulse-amplitude-modulated (PAM) chlorophyll fluorometer (MULTI-COLOR-PAM, Walz, Germany) [54]. After dark acclimation for 15 min at the culture temperature, measurements were taken at a light intensity of 50 µmol photons m^−2^ s^−1^, which was similar to the growth light level. Chlorophyll content and chlorophyll fluorescence data were analyzed using two-way ANOVA (GraphPad Prism).

### 4.4. Dry Biomass Measurement and Analysis of Protein, Total Carbohydrate, and Lipid Content

Dry biomass measurement: Samples were harvested after 14 days of cultivation. The wet biomass was centrifuged at 4000 rpm at 4 °C for 10 min and then washed twice with Milli-Q water to remove the salt. The cell pellets were lyophilized with vacuum freeze-drying equipment (FreeZone-18, Labconco, Kansas City, MO, USA) for 24 h. After drying, the cell pellets were weighed and stored at −80 °C until proteins, carbohydrates, and lipids were extracted and analyzed. 

For protein content, total protein was extracted from the lyophilized algal biomass and determined using the Bradford method (P0006C, Beyotime, Shanghai, China). For total carbohydrate content, total carbohydrates were determined by using a commercially available Total Carbohydrate Assay Kit (No. BC2715, Beijing Solarbio Science & Technology Co., Ltd., Beijing, China). For total lipid content, total lipids were extracted from the lyophilized algal biomass in chloroform–methanol (2:1, *v*/*v*). After centrifugation, the supernatant was collected into a new glass bottle, dried under a continuous stream of nitrogen gas, and then weighed. The above data were analyzed using *t*-test (GraphPad Prism).

### 4.5. Whole-Genome Sequencing and Analysis of Insertion Sites

Genomic DNA was extracted using the improved CTAB method, and total DNA was qualified and quantified using a NanoDrop and Qubit 2.0 fluorometer. Genomic DNA was sequenced using next-generation sequencing (NGS) by Novogene Co., Ltd. (Beijing, China); a library with an average 350 bp insertion size was constructed, and 2 × 150 bp paired-end sequencing was implemented on a NovaSeq 6000 system. Identification of insertion sites: Briefly, clean reads of filter sequencing data from *ECS* alignments were performed using the Burrows–Wheeler aligner (BWA) [55] using default parameters for paired-end reads and mapped to wild-type *N. oceanica* IMET1 genome version 2 (https://nandesyn.single-cell.cn/; accessed on 29 July 2022). The unmapped and mate unmapped reads were extracted and assembled into contigs using Spades [56]. Contigs with coverage depth below 20 were filtered (ref_unmapped), and plasmids containing Zeocin gene sequences were searched against ref_unmapped by BLAST [57]. The sites of the inserted fragment were determined using the E-value and the matching length. The insertion sites were manually inspected and visualized using Integrated Genomics Viewer (IGV) [58].

### 4.6. Confirmation of Insertion Sites

According to the insertion site results, specific primers were designed to detect insertion sites in the *ECS* genome. The primer sequences are presented in Supplementary Table S1. PCR was carried out by using 2× Accurate Taq Master Mix (dye plus) (Accurate Biotechnology Co., Ltd., Changsha, China) following the manufacturer’s instructions. A total of 50 µL of PCR amplification reaction volume contained 1 μL of DNA, 25 μL of 2× Tag mix, 22 μL of ddH_2_O, and 1 μL of 10 μM forward and reverse primers. The PCR program was initiated at 94 °C for 90 s, followed by 35 cycles of 98 °C for 10 s, 55 °C for 30 s, and 72 °C for 2 min.

Total RNA was extracted from the samples using the SteadyPure Universal RNA Extraction Kit (Accurate Biotechnology Co., Ltd., Changsha, China) according to the manufacturer’s instructions. Total RNA was qualified and quantified using a NanoDrop. cDNA was synthesized using the Evo M-MLV RT Kit with gDNA Clean for RT–qPCR (Accurate Biotechnology Co., Ltd., Changsha, China) following the manufacturer’s instructions. RT-qPCR was carried out by using an SYBR^®^ Green Premix Pro Taq HS qPCR Kit (Accurate Biotechnology Co., Ltd., Changsha, China) following the manufacturer’s instructions. A total reaction volume of 20 μL contained 1 μL of cDNA, 10 μL of SYBR Green (ROX mixed), 8.2 μL of ddH_2_O, and 0.4 μL of 10 μM forward and reverse primers. The PCR program was initiated at 95 °C for 20 s, followed by 40 cycles of 95 °C for 5 s and 60 °C for 30 s. mRNA levels were quantified using the 2^−ΔΔCT^ method [59]. Ubiquitin conjugating enzyme (*UBCE*) gene was used as an internal control. RT-qPCR data were analyzed using *t*-test (GraphPad Prism).

### 4.7. Identification and Characterization of Cyclin Genes in Nannochloropsis oceanica

Genomes, proteins, and gene structure annotation files of *N. oceanica* IMET1 were available at NanDeSyn (http://nandesyn.single-cell.cn/; accessed on 29 July 2022), and it was found to contain 10,333 genes. The cyclin protein sequence of *N. oceanica* IMET1 was identified through the following steps: First, hmmsearch [60] software was used with the hidden Markov model (HMM) for PF00134 (cyclin, N-terminal domain), PF02984 (cyclin, C-terminal domain), and PF08613 (cyclin, Pfam database, http://pfam.xfam.org/; accessed on 29 July 2022) to search the cyclin domain of the proteins of *N. oceanica* IMET1 proteomes, and the E-value threshold was set at 10^3^. Second, published cyclin proteins from *Arabidopsis thaliana*, *Oryza sativa*, *Nannochloropsis oceanica* CCMP1779, *Chlamydomonas reinhardtii*, and *Phaeodactylum tricornutum* were downloaded and used as query sequences to search against the *N. oceanica* IMET1 proteomes, and the E-value threshold was set at 10^5^. Third, the *N. oceanica* IMET1 annotation files were compared. Finally, the screening results of the three steps were merged, followed by manual removal of redundant or repetitive sequences. Prediction of the cyclin structure domain was carried out within the amino acid sequence of the candidate cyclin protein family member in *N. oceanica* IMET1 with the help of the Conserved Domain Database (CDD, http://www.ncbi.nlm.nih.gov/Structure/cdd/wrpsb.cgi; accessed on 29 July 2022) of the National Center for Biotechnology Information (NCBI, http://www.ncbi.nlm.nih.gov/; accessed on 29 July 2022), the Simple Modular Architecture Research Tool software (SMART, http://smart.embl-heidelberg.de; accessed on 29 July 2022), and the database of protein domains, families, and functional sites (Prosite, https://prosite.expasy.org/; accessed on 29 July 2022). Candidate genes without the cyclin structure domain were removed. The physicochemical properties of the cyclin protein of *N. oceanica* IMET1, including hydropathicity, molecular mass, instability index, and so forth, were predicted via ProtParam software from the Expasy database (http://web.expasy.org/protparam/; accessed on 29 July 2022).

### 4.8. Motif Analysis, Multiple Alignment, and Phylogenetic Analysis

The conserved protein motifs of the *N. oceanica* IMET1 cyclin protein sequences were identified using Multiple Em for Motif Elicitation (MEME) [60]. The analysis was performed using the following parameters: any number of repetitions, a maximum of 10 motifs, and optimum motif width from 10 to 60 amino acid residues. The conserved motifs were annotated using InterProScan (http://www.ebi.ac.uk/interpro/; accessed on 29 July 2022) [61]. Multiple alignment of the *N. oceanica* IMET1cyclin was performed using MAFFT [62], and the phylogenetic tree was inferred under maximum likelihood (RaxML) [63] with 1000 bootstrap replicates. The tree was visualized using EvolView (https://www.evolgenius.info/evolview/; accessed on 29 July 2022) [64].

To comprehensively understand the evolutionary relationships of cyclin members, a phylogenetic tree was generated based on *Chlamydomonas reinhardtii* cyclin (Chlre5) proteins, *Nannochloropsis oceanica* CCMP1779 cyclin (No1779) proteins, and *Phaeodactylum tricornutum* cyclin (Phatr2) proteins, especially *Oryza sativa* cyclin (Orysa) and *Arabidopsis thaliana* cyclin (Arath) proteins, as references to categorize the *N. oceanica* IMET1 cyclin proteins.

### 4.9. Transcriptomic Data Analysis

To gain insight into the expression profile of *N. oceanica* IMET1 cyclin genes, transcriptomic data were analyzed during the light/dark cycles. We downloaded transcriptomic data of PRJNA285666 (light/dark cycles) from the NCBI database. The expression levels of *N. oceanica* IMET1 cyclin genes were quantified based on their fragments per kilobase of transcript per million mapped reads (FPKM). Briefly, using the *N. oceanica* IMET1 genome (IMET1v2) as the reference genome, the example transcriptomic data were aligned to the reference genome with TopHat [65], and gene expression was measured as the number of reads aligned to annotated genes through Cufflinks [66] and normalized to FPKM. The FPKM values were log2-transformed and used to construct a heatmap.

### 4.10. Cell Cycle Analysis Using Flow Cytometry

The cells were cultured to the middle of the logarithmic phase, and Zeitgeber time 0 (ZT0) was used as the starting point. Two samples were collected every 3 h, and one sample was moved to a dark environment and cultured for 24 h. The ZT0 samples were collected in the dark, and the ZT12 samples were collected in the light. The cells were centrifuged at 4000× *g* for 5 min at 4 °C, and the cell pellet was fixed overnight in 70% ethanol at 4 °C, washed twice in phosphate-buffered saline (PBS) at pH 7.5, and stained in PBS 0.1% Triton-X and 1 μg mL^−1^ of 4′,6′-diamidino-2-phenylindole (DAPI, MBD0015, Sigma-Aldrich, USA) for 20 min. Flow CytoFLEX S flow cytometry analysis of the cell cycle was performed using a 375 nm ultraviolet (UV) light laser and a 450/45 bandpass filter, and 20,000 cells were analyzed per sample. FlowJo (v10.8.1) was used for data analysis.

### 4.11. RT–qPCR Analysis of N. oceanica IMET1 Cell-Cycle-Related Genes

To measure the expression of cell-cycle-related genes in cultured cells across a 24 h day–night cycle, the cells were cultured to the middle of the logarithmic phase and, at ZT0 as the starting point, were harvested every 3 h by centrifugation, washed twice in Milli-Q water, flash-frozen in liquid nitrogen, and stored at −80 °C. For each time point, three biological repeats were performed. Total RNA was extracted, cDNA was synthesized, and gene expression analysis was performed using the RT-qPCR referenced in Materials and Methods, Section 2.4. The primer sequences are presented in Supplementary Table S1. All experiments were performed with three biological repeats, with two technical repeats generated for each one.

## 5. Conclusions

In the present study, we observed that *ECS* cell enlargement significantly decreased cell density, and increased Fv/Fm, chlorophyll a content, cell dry weight, and protein and lipid content per cell. Furthermore, we found and demonstrated that the insertion fragment was integrated inside the 5′-UTR of the U/P-type cyclin *CYCU;1*, which is a highly expressed cyclin. Furthermore, *ECS* could not fully complete cell division in the dark phase, and the expression level of *CYCU;1* increased significantly at the end of the light phase and the beginning of the dark phase, indicating that this cyclin regulates cell division in the dark phase. Our results explain the reason for *ECS* cell enlargement and reveal that *CYCU;1* controls cell size and cell density by regulating cell division, providing a target gene for genetic engineering to regulate microalgae cell density.

## Figures and Tables

**Table 1:** Characteristics of cyclin genes identified in *Nannochloropsis oceanica*.

Gene Name	Gene ID in N.o IMET1	Locus in Genome	Gene Features		Protein Features					Pfam (Position)	SMART (Position)	CDD (Position)	Prosite (Position)
			CDS Length	No. of Introns	Protein Length	*p*I	MW	Instability Index	GRAVY				
*CYCL;1*	NO01G01210	chr1:315,224-316,537	1314	0	437	9.48	48,434.27	60.81	−0.516	PF00134 (135-204)	SM00385 (138-246)	cd00043 (137-192)	
*CYCH;1*	NO02G04130	chr2:1,126,722-1,128,367	1185	1	394	6	43,296.16	51.85	−0.378	PF00134 (142-214) PF16899 (218-308)	SM00385 (115-208)	cd00043 (141-207)	
*CYCT;1*	NO02G05390	chr2:1,437,666-1,439,100	1050	1	349	7.03	39,336.56	54.86	−0.445	PF00134 (26-106)	SM00385 (29-181, 194-336)	cd00043 (31-110)	
*CYCP;1*	NO03G03610	chr3:1,070,086-1,072,470	2199	0	732	5.18	82,325.05	74.32	−0.839	PF00134 (401-446)		cd00043 (398-445)	
*CYCL;2*	NO03G03880	chr3:1,139,762-1,141,883	1779	0	592	6.03	64,464.14	68.61	−0.65	PF00134 (71-206)	SM00385 (80-198, 217-299)	cd00043 (74-169, 214-298)	
*CYCP;2*	NO03G05460	chr3:1,562,402-1,566,971	2340	4	779	5.94	86,517.33	69.46	−1.086	PF08613 (371-677)	SM00385 (576-672)	cd20540 (572-675)	
*CYCD;2*	NO05G01050	chr5:313,215-314,347	960	1	319	9.22	34,130.23	57.4	−0.086	PF00134 (52-145) PF02984 (154-231)	SM00385 (59-145) SM01332 (98-248)	cd00043 (54-143)	PS00292 (54-85)
*CYCD;1*	NO07G04620	chr7:1,302,238-1,305,696	2487	2	828	9.25	86,631.02	66.54	−0.358	PF00134 (474-592) PF02984 (603-739)	SM00385 (505-590, 607-705) SM01332 (603-740)	cd00043 (499-589, 603-662)	
*CYCP;3*	NO10G00130	chr10:40,340-43,308	1539	10	512	7.75	58,056.13	74.76	−0.77	PF00134 (328-433)	SM00385 (341-426)	cd00043 (339-424)	
*CYCB;1*	NO12G01680	chr12:502,593-504,248	1416	2	471	5.61	51,922.13	62.33	−0.29	PF00134 (227-350) PF02984 (354-465)	SM00385 (260-344, 357-438) SM01332 (353-468)	cd00043 (254-343, 351-436)	PS00292 (255-286)
*CYCP;4*	NO12G03480	chr12:951,333-954,950	1953	1	650	6.3	71,251.35	72.85	−0.641	PF00134 (333-439)	SM00385 (349-434)	cd00043 (347-433)	
*CYCA;1*	NO13G01690	chr13:499,826-502,404	1650	1	549	7.57	60,194.35	67.26	−0.412	PF00134 (274-402) PF02984 (405-534) PF08613 (371-677)	SM00385 (312-396, 409-506) SM01332 (405-537)	cd00043 (306-395, 406-504)	
*CYCU;1*	NO14G02130	chr14:640,825-642,525	1701	0	566	8.23	61,891.97	67.84	−0.483	PF08613 (47-159)	SM00385 (69-153)	cd20558 (63-162)	
*CYCL;3*	NO15G01200	chr15:342,170-343,339	939	2	312	9.31	34,799.32	46.11	−0.265	PF00134 (16-139)	SM00385 (33-132)	cd00043 (28-105)	
*CYCA;2*	NO19G00890	chr19:301,869-304,149	2211	1	736	4.72	82,335.81	60.24	−0.593	PF00134 (145-268)	SM00385 (175-261)	cd00043 (169-260)	
*CYCL;4*	NO22G00250	chr22:89,639-94,300	1677	9	558	8.74	60,996.24	73.95	−0.673	PF00134 (150-269)	SM00385 (163-263)		
*CYCB;2*	NO23G01320	chr23:476,595-480,938	2532	6	843	5.59	89,728.7	90.25	−0.666	PF00134 (524-630)	SM00385 (537-625, 652-743) SM01332 (634-778)	cd00043 (531-563	PS00292 (532-563)
*CYCB;3*	NO25G01730	chr25:508,155-511,392	1662	6	553	5.78	59,436.75	56.39	−0.45	PF00134 (301-422) PF02984 (425-541)	SM00385 (332-416, 429-510) SM01332 (425-545)	cd00043 (326-415, 425-508)	PS00292 (327-358)
*CYCU;2*	NO28G01340	chr28:421,745-424,038	933	3	310	9.31	34,082.29	72.84	−0.352	PF08613 (173-282)	SM00385 (190-276)		PS00292 (185-216)

## Data Availability

The whole-genome-sequencing clean reads of *ECS* and WT were deposited in the GenBank SRA database under accession number PRJNA1001626.

## Supplementary Materials

The following supporting information can be downloaded at https://www.mdpi.com/article/10.3390/ijms241713595/s1.
