# Supplementary material for: Phenotypic Analysis and Molecular Characterization of Enlarged Cell Size Mutant in Nannochloropsis oceanica
Source: Int J Mol Sci. 2023 Sep 2;24(17):13595. doi: 10.3390/ijms241713595 (PMC10487731; doi:10.3390/ijms241713595)
Supplement: Supplementary file 1 [file ijms-24-13595-s001.zip › supplementary_Figure.pdf]

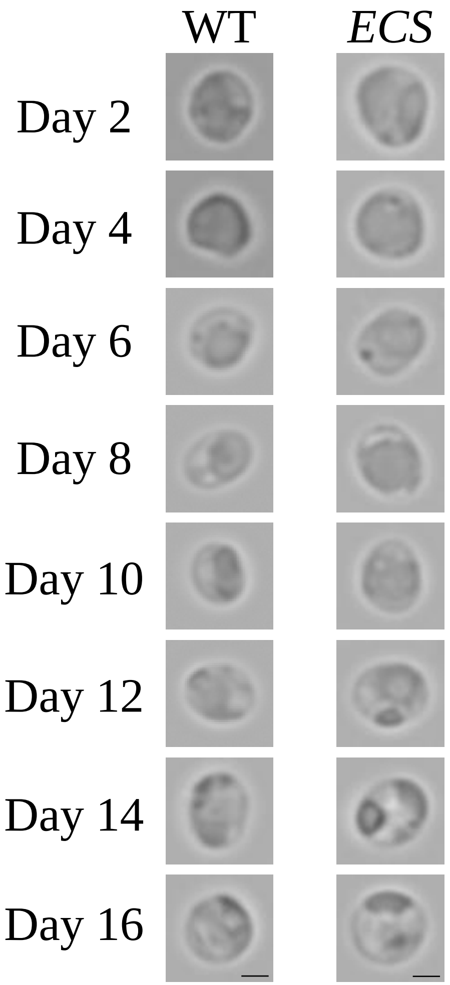

Figure S1. Cell area of WT and *ECS*. Select a cell whose cell area is close to the average in Table S2. Bar = 1 μm

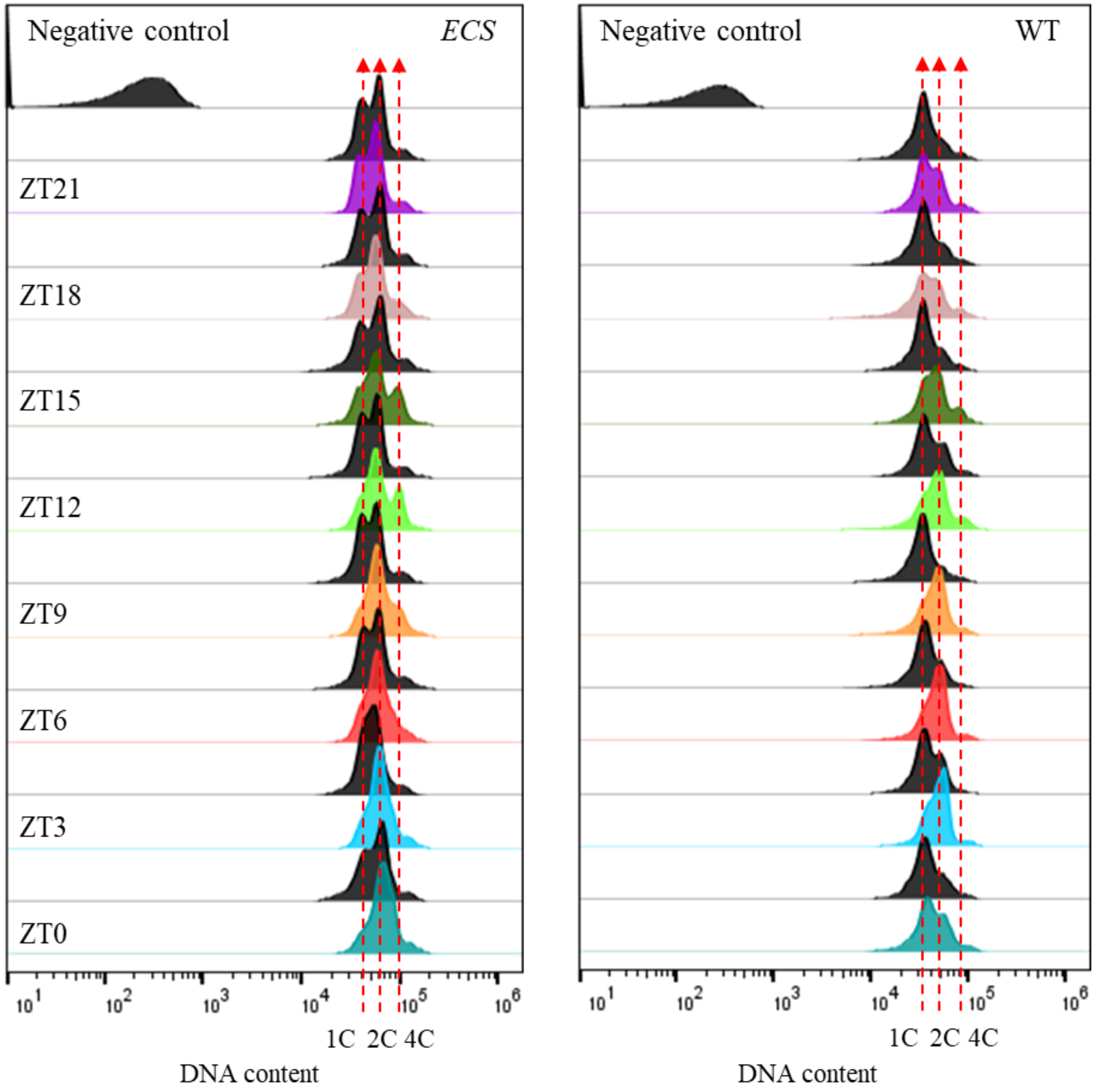

Figure S2. Flow cytometry analysis cell cycle. DNA content per cell measured as DAPI fluorescence using flow cytometry at different times of day (ZT, hours after lights on), dark color means sample additional 24 hours of dark culture.
